# Account of Diseases in London, from the 20th of March to the 20th of April

**Published:** 1799-05

**Authors:** 


### *Account of Diseases in London, from the 20th of March to the 20th of April.*

| | No. fCases. |
|---|---|
| ACUTE DISEASES. | |
| Typhus Gravior | 3 |
| Typhus Mitior | 5 |
| Peripneumonia | 3 |
| Peripneumonia Notha | 5 |
| Catarrh | 4 |
| Inflammatory Sore-throat | 3 |
| Ophthalmia | 1 |
| Acute Rheumatism | 6 |
| CHRONIC DISEASES. | |
| Cough | 12 |
| Dyspnœa | 7 |
| Cough and Dyspnœa | 13 |
| Hoarseness | 4 |
| Hæmoptoe | 3 |
| Phthisis Pulmonalis | 10 |
| Pleurodyne | 4 |
| Ascites | 2 |
| Anasarca | 3 |
| Cephalalgia | 10 |
| Syncope | 3 |
| Vertigo | 5 |
| Epistaxis | 2 |
| Menorrhagia difficilis | 4 |
| Amenorrhœa | 7 |
| Chlorosis | 5 |

| | No. of Cases. |
|---|---|
| Fluor albus | 6 |
| Enterodynia | 5 |
| Diarrhœa | 9 |
| Vomitus | 3 |
| Gastrodynia | 4 |
| Dyspepsia | 10 |
| Obstipatio | 2 |
| Hæmorrhois | 3 |
| Prolapsus Ani | 1 |
| Dysuria | 3 |
| Herpes | 4 |
| Hysteria | 3 |
| Palpitation of the Heart | 2 |
| St. Vitus's Dance | 2 |
| Hypochondriasis | 3 |
| Chronic Rheumatism | 14 |
| PUERPERAL DISEASES. | |
| Dolores post Partum | 3 |
| Menorrhagia lochialis | 2 |
| Low Fever | 2 |
| Mastrodynia | 3 |
| INFANTILE DISEASES. | |
| Aphthæ | 4 |
| Ophthalmia | 3 |
| Fever | 2 |
| Vermis | 4 |

The unusually long continuance of cold weather, and the pretty constant course of the wind from the East, and North-East, have protracted the duration of many of the winter diseases. Cough, dyspnœa, catarrh, and all the species of pneumonic affections, still continue to prevail very generally. In several cases of phthisis pulmonalis, the symptoms have seemed to be aggravated by the state of the weather: and there has been a very rapid advance towards the fatal catastrophe.

Rheumatisms, both of the acute and chronic species, have been very frequent. In this disease, the degree of pain has been very considerable, and the recovery from the acute species of it uncommonly slow. Some patients who had recovered from acute rheumatism, previously to the commencement of the winter, and who during the first of the winter months, continue from every symptom of the disease, have been much afflicted by the wandering pains of the chronic species, during the last two months.

Inflammatory sore-throats have been very frequent, and have been followed, especially in scrophulous habits, by a tenderness and enlargement of the parotid glands.

